# Comparative analysis of humoral immunity kinetics following three COVID-19 vaccines in a multi-ethnic cohort of medical students and healthcare professionals across Malaysia

**DOI:** 10.1038/s41598-025-07895-6

**Published:** 2025-07-01

**Authors:** Ashwathy Varadarajan Thundakattil, Rekha Prabhu, Girish Prabhu, Madhumanti Barman, Muthuvel Mani, Somsubhra De, Htay Lwin, Nelli Giribabu, Sabyasachi Das

**Affiliations:** 1 Department of Physiology, Faculty of Medicine, Manipal University College Malaysia, Melaka, Malaysia; 2 Department of Biochemistry, Faculty of Medicine, Manipal University College Malaysia, Melaka, Malaysia; 3 Department of Physiology, Faculty of Medicine, Universiti Malaya, Kuala Lumpur, Malaysia; 4 Department of Anatomy, Faculty of Medicine, Manipal University College Malaysia, Melaka, Malaysia; 5 Department of Obstetrics and gynaecology, Faculty of Medicine, Manipal University College Malaysia, Melaka, Malaysia; 6 Department of Community Medicine, Faculty of Medicine, Manipal University College Malaysia, Melaka, Malaysia; 7 Department of Physiology, Faculty of Medicine, University Malaya, Kuala Lumpur, Malaysia

**Keywords:** Comparative antibody response, Comirnaty vaccine, Vaxzevria vaccine, CoronaVac vaccine, Time dependent response, Ethnicity, Waning of antibody, Malaysia, Antibody generation, ELISA, DNA vaccines, Inactivated vaccines, RNA vaccines

## Abstract

The objective of our study was to evaluate the time-dependent humoral immunity kinetics among multi-ethnic population of medical students and healthcare professionals across Malaysia following two doses of Comirnaty, Vaxzevria, and CoronaVac vaccines. Prospective observational cohort research was carried out from September 2021-March 2023. 242 vaccine recipients have completed the follow-up. After second vaccination dosage, peripheral blood was drawn every four weeks from week 0–24. Anti-S IgG and percentage of neutralization were measured from each individual plasma sample. Recipients of Comirnaty and Vaxzevria vaccine, exhibited adequate anti-S IgG and neutralizing antibody till week 24. In compared to Comirnaty/Vaxzevria vaccine group, CoronaVac group showed significant decrease of antibody as early as week 12. The Comirnaty vaccine peaked anti-S IgG production at week 4, while the Vaxzevria and CoronaVac vaccines peaked at week 2. The Comirnaty vaccine (91.3%) showed strong Ig-RBD neutralizing antibody response even after week 24, which led to the slowest rate of antibody waning when compared to CoronaVac (13%), which never achieves high Ig-RBD neutralizing antibody. The percentage of inhibition of Ig-RBD binding on ACE_2_ receptor and anti-S IgG seroconversion were significantly higher in the Comirnaty vaccine. The antibody response of Comirnaty was lower in recipients with higher BMIs than in those with normal or low BMIs. Higher BMI, however, was associated with more robust humoral immune responses to Vaxzevria vaccine. Following immunisation, the Chinese and Indian populations might exhibit a stronger humoral immune response than the Malay population.

**Supplementary Information:**

The online version contains supplementary material available at 10.1038/s41598-025-07895-6.

## **Introduction**

Since 2020, the new COVID-19 infection has had a disastrous impact on people all around the world. In Malaysia, the first case of COVID-19 was reported on 25 January 2020^1^. The Malaysian Ministry of Health (MOH) reported a total of 5,278,406 COVID-19 cases and 37,348 deaths from February 2020 to April 2024^2^. Four outbreaks in Malaysia were caused by the COVID-19 variants Wuhan, Beta, Delta, and Omicron. The Wuhan variant outbreak lasted for 29 weeks, from January to August 2020^3^. The beta version outbreak lasted for 30 weeks, from September 2020 to March 2021^4^. The Delta outbreak lasted for forty-three weeks, from April 2021 to January 2022^5^. The Omicron outbreak lasted from January 2022 to April 2022, when the illness was deemed endemic^6^. The Delta and Omicron outbreaks had the highest number of COVID-19 cases, attributable to the prolonged outbreak duration, greater population morbidity, and increased mortality rates^7^. Intense global efforts have resulted in the creation of multiple SARS-CoV-2 vaccination platforms, including viral vectored, mRNA, DNA, recombinant protein, and live attenuated vaccines to limit the mortality and morbidity^8^. Following the necessary approval from the MOH, three COVID-19 vaccinations are commonly administered in Malaysia as part of the country’s immunisation program for the general public. These vaccines include the inactivated COVID-19 vaccine CoronaVac, the mRNA vaccine Comirnaty, and the single recombinant vaccine Vaxzevria^9^. As of 26th of November 2023, 85% of total Malaysian population was vaccinated with primary series (two doses) of a COVID-19 vaccine^2,10^.

Despite the fact that many vaccines were created using various techniques, all or most of the vaccines have the fundamental objective of boosting humoral immunity through generating COVID-19 spike (S) protein-specific antibodies in humans, more precisely IgG and IgM antibodies^11–14^. The novel SARS-CoV-2 virus produces a number of structural proteins, including the envelope (E), membrane (M), nucleocapsid (N), and Spike (S) protein. However, in the absence of anti-S antibodies, antibodies against N, M, and E do not neutralise the virus^15^. The neutralization of novel corona virus is predominantly depending on the magnitude of immunoglobulin-receptor binding domain (Ig-RBD) antibody and the proportion of plasma B-cells, Memory B-cells and T-cells that are activated^16^.

Health care professionals were the most affected group in the contemporary civilized society during the COVID-19 pandemic and had the highest risk of acquiring a new infection. They are thus the first group of individuals in Malaysia to receive the early dosage of the vaccination. Previous research has demonstrated that a number of factors, including the type of vaccination, the interval between doses, age, ethnicity, body type, dietary habits, etc., affect the formation of antibodies^16–20^. Several studies have also demonstrated that different ethnicity may also play crucial role in responses to immunogenicity following vaccinations^19,21^. –^22^ Malaysia is a multi-ethnic country^23^, therefore evaluating the immune response following various vaccinations could provide important insights into the duration-dependent effectiveness of vaccines.

The knowledge about waning antibody response following monovalent or bivalent homologous vaccination against novel COVID-19 and SARS-CoV-2 mutant serotypes were of utmost importance. However, duration dependent precise antibody kinetics following two doses of three different vaccines across Malaysia remains unknown^24^.

This study, therefore aimed to prospectively compare the antibody responses (humoral immunity) of two doses of the Comirnaty, Vaxzevria, and CoronaVac vaccines among multi-ethnic healthcare professionals and medical students in Malaysia.

## Materials and methods

### Study design and selection of participants

#### Study design

This multicentric prospective observational cohort study was conducted to evaluate the humoral immunity following Comirnaty, Vaxzevria, and CoronaVac vaccinations among the healthcare professionals and medical students of Manipal University College Malaysia (MUCM) (Melaka and Muar campus), and University of Malaya (UM), Malaysia from September 2021 to March 2023. During our study period Malaysia experienced Delta and Omicron variant outbreaks. We were unable to include unvaccinated people (non-exposure group) in our study population since the Ministry of Health, Government of Malaysia, mandated vaccination for all federal government employees and healthcare professionals in September 2021^25^. Before inception of the study, participants had to fill out a patient information sheet and informed consent form for donating of biological samples. A total of 503 individuals underwent screening. The experimental design and methods employed in this investigation were duly approved by the Manipal University College Malaysia, Melaka human ethics committee (MUCM/FOM/Research Ethics committee − 5/2021), and the experimental protocol of this study complied with the guidelines set forth by the Ministry of Health of Malaysia (MOH). Hypertension, BMI, fasting blood sugar, HbA1c etc.) were assessed to identify the baseline characteristics of the vaccine recipients. Detailed methods are mentioned in supplementary document.

#### Inclusion criteria

Unexposed adults, never infected with SARS-CoV-2 nor vaccinated against COVID-19 were included for the study following confirmation through Govt of Malaysia My Sejahtera application and cross-verified by serologic assessment of anti-S IgG antibody^26,27^. Depending on their personal preferences, study participants received either Comirnaty, Vaxzevria, or CoronaVac vaccine intramuscularly. For Comirnaty and CoronaVac, the time between the first and second doses of the vaccine was 21 days, whereas for Vaxzevria, it was 56 days. A core body temperature of less than 37.5⁰C and an age more than 18 years were prerequisites for the inclusion in the study.

#### Exclusion criteria

Participants were eliminated from the study if they had been infected with COVID-19 within the previous three months. Seropositive COVID-19 infection during the 24-weeks follow-up was also excluded. It was verified by the Malaysian government My Sejahtera application and crosschecked through RT-PCR based detection of COVID-19 infection^27,28^. Individuals who experienced serious side effects after immunisation and needed hospitalization were excluded. Individuals who were known to have a history of autoimmune disease, an immunocompromised state, were not allowed. Mixed ethnic participants were not also included in this study.

#### Precautions taken to limit bias

Malaysia is a multi-ethnic society with three main ethnicities namely the Malays, Chinese and Indians. Around 70.4% of the Malaysian population were classified as Malays, 22.4% were classified as ethnic Chinese, and 6.5% as ethnic Indians. Vaccine recipients from all three ethnic groups of Malaysia were recruited to ensure the multi-ethnic representation. The characteristic or reality of being a member of a demographic group or subgroup composed of individuals with similar social backgrounds, cultural practices, and eating habits. We have differentiated the three ethnic groups depending on their family history of since last 5 generation, and their morphological differences. Samples of peripheral blood were duly collected at predetermined intervals of 0, 2, 4, 8, 12, 16, and 24 weeks after second dose of vaccine. To limit the individual biasness, all the collected samples and individual recipients’ details were blindly coded.

### Collection of blood samples and isolation of plasma

Peripheral blood samples (1 ml) were collected intravenously at week zero (before the first dose), week two (W2), week four (W4), week 8 (W8), week 12 (W12), week 16 (W16), week 20 (W20), and week 24 (W24) following 2nd dose of vaccination as per the Helsinki protocol. Blood samples were kept in 1.5 ml anti-coagulant (EDTA) coated micro centrifuge tube as per our standard laboratory protocol^29^. Plasma was isolated from the collected blood sample using Histopaque-1077 density gradient by centrifugation at 320 X g for 45 min. Following centrifugation, the topmost layer of plasma was collected and stored at -80 °C in a 1.5 ml micro centrifuge tube^29^.

### Body mass index

Previous report suggested that a higher BMI may have decreased the humoral response to SARS-CoV-2 infection^30^. Therefore, we assessed individual BMI of the vaccine recipients. An electronic weighing machine was used to assess individual body weight during a monthly follow-up while they were wearing loose-fitting clothes and no shoes. A “drop-down” tape was used to determine height. The weight (kg) of each recipients was divided by the square of their height (m^2^) to get their BMI.

### Estimation of COVID Spike (S) protein specific plasma IgG

We estimated the COVID S-antigen specific anti-S IgG antibody from individual plasma samples by ADVIA Centaur SARS-CoV-2 spike IgG assay kit (Siemens Healthcare Diagnostic, NY, USA) following manufacturers instruction. Samples were analysed in duplicate and their geometric mean was taken. Anti-S IgG was quantitatively detected using a two-step sandwich immunoassay^26^. The assay result was presented as sCOVG index value (U/ml), which was then translated into binding antibody units (BAU) per millilitre (BAU/ml), the WHO standard for anti-SARS-CoV-2 immunoglobulin unit^31^. The conversion formula is sCOVG index value (U/ml) X 21.8 = BAU/ml. The seropositive cut-off was 5U/ml equivalent to 109 BAU/ml.

### Estimation of neutralizing antibody

Following the manufacturer’s instructions, neutralizing antibodies (Ig-RBD) that targeted the novel SARS-CoV-2 virus’s ACE_2_ receptor. A02161 was utilized as the positive control when the SARS-CoV-2 Surrogate Virus Neutralization Test (sVNT) Kit (GenScript, Cat.No. L00847-A) was administered with Omicron RBD-HRP (Z03730). The assay result was expressed as percentage (%) of inhibition of RBD binding on angiotensin-converting enzyme 2 (ACE2). More than 90% inhibition was considered high neutralization, 60–90% inhibition was considered as medium neutralization, and 30–60% inhibition was considered weak neutralization. Inhibition rates of less than 30% were considered non-neutralizing^32^. Initial sample dilution factor was 1:1, followed by 1:2 dilution using RBD-HRP. Neutralizing antibody was tested for all vaccine recipients. Optical density was measured as 450 nm.

### Statistical analysis

The data was reported as mean ± SD. The Clopper-Pearson exact technique was used to calculate the 95% confidence interval (CI) for age and mean anti-S IgG antibody. We had performed Shairo-Wilk test to check the normality distribution of data. The anti-S IgG titre was compared between two groups at each time point using the Mann-Whitney U-test. Kruskal-Wallis H test was used to evaluate the differences in antibody titre among various ethnic groups. The Spearman rank correlation test was used to analyse the correlation between neutralizing Ig-RBD and Anti-S IgG. *P* < 0.05 was taken into consideration for significant statistical differences. Graph Pad in Stat software 3.0 and Origin 6.1, a statistical tool, were used to conduct the statistical study.

## Results

### Baseline characteristics of vaccine recipients

**Fig. 1:**
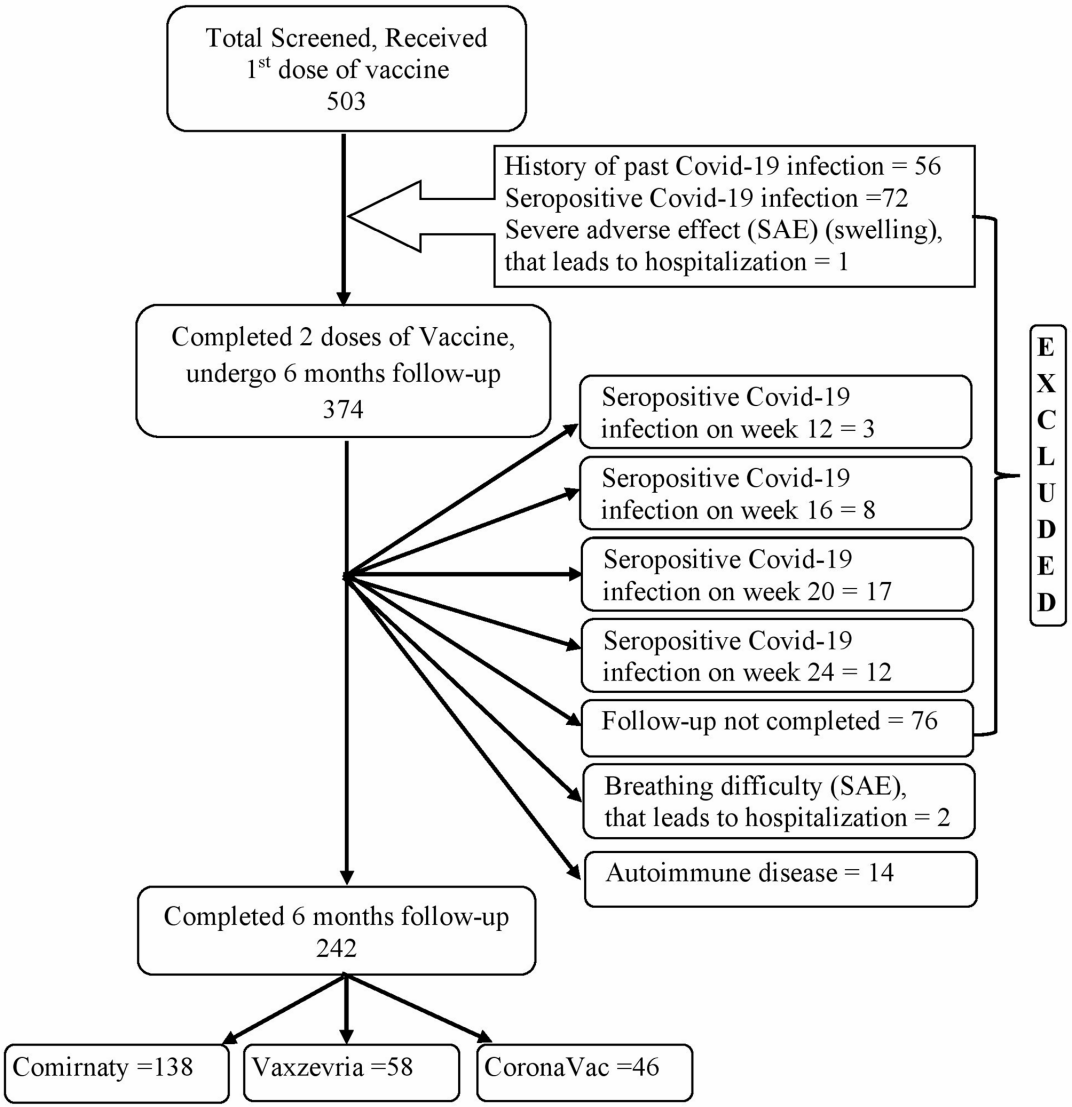
Strobe diagram for selection of study participants.

A total of 503 people were screened, of them 242 (48.1%) recipients completed the follow-up. A total of 56 recipients (11.1%) were excluded due to their history of COVID-19 infection within the previous three months. Additional 72 (14.1%) recipients were excluded as they were found to be seropositive COVID-19 infections between 1st and 2nd dose of vaccination. Majority (76.4%) of vaccine recipients who developed seropositive COVID-19 infection had received CoronaVac. After the second dose of the vaccination, 40 (7.9%) recipients had suffered with seropositive COVID-19 infection within 24 weeks. The majority of these seropositive COVID-19 infections (83.8%) were vaccinated with CoronaVac, followed by Vaxzevria (13.5%) and Comirnaty (2.7%). Figure 1 listed the precise number of patients who tested positive for COVID-19 at a certain time point. Three (0.6%) recipient developed severe adverse effects (difficult of breathing to two person and one person had sever swelling welling of the face, lips, eye and throat) followed by hospitalization were eliminated from the study. Another 14 (2.8%) recipients were eliminated as they were suffering from different autoimmune diseases (rheumatoid arthritis-4, systemic lupus erythematosus-3, psoriasis-3, ankylosing spondylosis-2, multiple sclerosis-1, irritable bowel syndrome-1,). Remaining 76 (15.1%) recipients had not completed the follow-up. Detailed characteristics of the recipients are presented in Table 1. Out of 242 vaccine recipients 138 (57%) individuals received Comirnaty followed by 58 (24%) individuals received Vaxzevria and remaining 46 (19%) participants received CoronaVac vaccines. The mean age of the vaccine recipients was 28.4 years (ranging from 18 to 64). The mean age of Comirnaty vaccine recipients [35.6(± 14.3)] was substantially (*P* < 0.001) higher than that of Vaxzevria recipients [22.8 (± 5.1)] and CoronaVac recipients [20.4 (± 2.4)]. The demographic characteristics of the in our study were 20.3% Malays, 38.8% Chinese and 40.9% Indians. The ethnic composition of the recipients of the three different vaccines did not differ statistically significantly (*P* = 0.518), even though more than 52% of the CoronaVac group and 50% of the Vaxzevria vaccine group were Chinese and Indian, respectively.

**Table 1:** Baseline characteristics of vaccination study population.

Recipient details	Type of vaccination			*P* value
	Comirnaty (	Vaxzevria	CoronaVac	
Interval between dose 1 and 2 vaccine	21 days	56 days	21 days	
No of recipients	138	58	46	
Age^a^	35.6 (± 14.3)	22.8 (± 5.1)	20.4 (± 2.4)	< 0.001
Shapiro-Wilk test *P* value for age	0.705	0.0576	0.0643	
BMI^b^ (kg/m^2^)				
< 18	8 (5.8%)	8 (13.8%)	6 (13%)	0.960
18–24.99	72 (52.1%)	30 (51.7%)	22 (47.8%)	
25–29.99	34 (24.6%)	8 (13.8%)	12 (26.1%)	
30–34.99	18 (13%)	6 (10.3%)	6 (13%)	
35–39.99	6 (4.3%)	6 (10.3%)	0	
Ethnicity^b^				
Malay	30 (21.7%)	9 (15.5%)	10 (21.7%)	0.518
Chinese	50 (36.2%)	20 (34.5%)	24 (52.2%)	
Indian	58 (42%)	29 (50.0%)	12 (26.1%)	
Gender^b^				
Male	44 (31.9%)	19 (32.8%)	16 (34.8%)	0.939
Female	94 (68.1%)	39 (67.2%)	30 (65.2%)	
Female to male ratio	2.1	2.0	1.9	
Co-morbidity^b^				
Hypertension	25 (18.1%)	4 (6.9%)	1 (2.2%)	0.3663
Type-2 diabetes	15 (10.9%)	1 (1.7%)	2 (4.3%)	
Chronic respiratory disease	7 (5.1%)	5 (8.6%)	3 (6.5%)	
Coronary artery disease	4 (2.9%)	0 (0.0%)	0 (0.0%)	
Liver disease	2 (1.5%)	2 (3.4%)	0 (0.0%)	

^a^*P* value was obtained using one-way Anova.

^b^*P* value was calculated using chi-square test.

The majority of recipients were female (67.4%), and as a result, there was a very high female to male ratio in all vaccination groups. The majority of recipients (70.7%) were in good health and did not have any coexisting medical conditions. Hypertension (12.4%), diabetes (7.4%), and chronic respiratory disease (6.2%) were the most often reported co-morbidities (Table 1).

### Seropositive anti-S IgG antibody kinetics

Comparative analyses of time dependent post-vaccination serological titres of anti-S IgG with their cut-off response were presented in Table 2. Regardless of ethnicity, in contrast to the CoronaVac group, the Comirnaty and Vaxzevria groups showed sufficient levels of anti-S IgG antibodies from W2 to W24 following the second dose of vaccine. Of the Comirnaty group, not even a single subject had anti-S IgG levels below the threshold (109BAU/ml) until W12. Conversely, in the CoronaVac group, 13.0% of individuals on W8 had anti-S IgG levels below the positive threshold, and this number increased to 54.3% in W16. Merely 1.7% of recipients from the Vaxzevria group were found to have less anti-S IgG levels in W8 and W12, respectively (Table 2). Compared to the CoronaVac group, the Comirnaty vaccine group had considerably (*P* = 003) lower number of recipients with below threshold anti-S IgG antibodies (Table 2) at W12. At W16 and W24 after 2nd Comirnaty vaccination only 0.7% and 2.9% of individuals had anti-S IgG antibodies below the positive cut-off, whereas 54.3% and 84.8% of recipients in the CoronaVac group had anti-S IgG antibodies below the threshold. However, anti-S IgG were found to be reduced to some extent in W16 (6.9%) and W24 (12.1%) following Vaxzevria vaccination (Table 2). Even though female subjects were more there was no significant difference of anti-S IgG and gender among the three vaccine groups (Comirnaty, *P* = 0.216, Vaxzevria, *P* = 0.079, CoronaVac *P* = 0.165).

**Table 2:** Anti-S IgG seroconversions to Comirnaty, Vaxzevria and Coronavac vaccine recipients.

Type of vaccine	No.	Anti-S IgG seroconversions (values in percentage %)						
		W2	W4	W8	W12	W16	W20	W24
Comirnaty–Comirnaty	138	100.0	100.0	100.0	100.0	99.3	98.6	97.10
Vaxzevria–Vaxzevria	58	94.8	100.0	98.3	98.3	93.1	91.4	87.9
CoronaVac–CoronaVac	46	100.0	100.0	86.9	69.6	45.7	34.8	15.2

Seropositivity anti-S IgG antibody cut off is 5 U/ml which equivalent to 109 BAU/ml.

### Duration dependent comparative anti-S IgG response and ethnicity

**Fig. 2:**
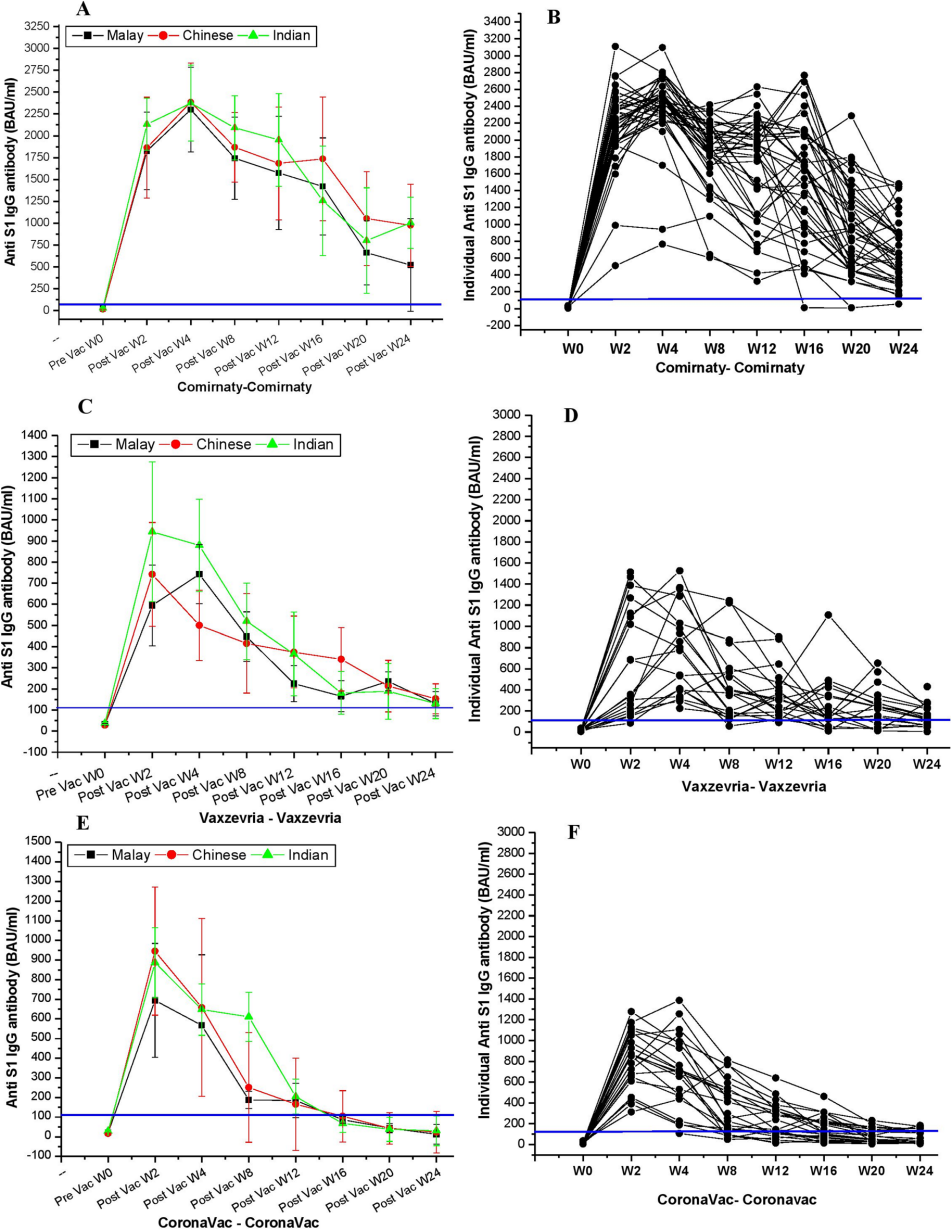
Kinetics of anti-S IgG antibody. (**A**) The changes in the mean anti-S IgG antibody kinetics among different ethnic groups of Malaysia following the administration of the two doses of the Comirnaty vaccination. (**B**) The kinetics of individual anti-S IgG antibody after Comirnaty vaccine. (**C**) The alteration of mean anti-S IgG antibody over time following Vaxzevria vaccination. (**D**) The individual anti-S IgG kinetics after Vaxzevria vaccine. (**E**) The anti-S IgG antibody kinetics following CoronaVac vaccination. (**F**) The individual anti-S IgG antibody kinetics after CoronaVac vaccine. The blue line hypothetically represented the positive threshold (109 BAU/ml) of anti-S IgG antibody.

Following W2, seropositive anti-S IgG responses are developed in 100%, 94.8%, and 100% of Comirnaty, Vaxzevria, and CoronaVac recipients, respectively. Regardless of race, the peak of the antibody response after the Comirnaty vaccination was reached at W4, while the peak of the antibody response following the Vaxzevria (except from the Malay population) and CoronaVac vaccinations was found at W2 (Fig. 2A, C and E). We found that anti-S IgG seroconversions among different ethnic group were not normally distributed (Shapiro-Wilk test *P* = 0.0019 for Chinese and India, *P* = 0.012 for Malay). The Kruskal-Wallis H test revealed a statistically significant difference (*P* < 0.0001) between the mean antibody response of Comirnaty, Vaxzevria, and CoronaVac. In W2, following Comirnaty vaccination, the anti-S IgG antibody level in Indian population (2132.6 BAU/ml, 95%CI, 1760–2346) was substantially greater (*P* = 0.008) than the Chinese populations (1865.1 BAU/ml, 95%CI 1630–2110) and Malay (1822.3 BAU/ml, 95%CI 1670–2063) populations (Fig. 2A). The antibody response in W4 was increased in all ethnic (Malay, Chinese, and Indian) populations and reached as high as 2300, 2385, and 2373 BAU/ml, respectively. After the W4 of the second Comirnaty vaccine, a steady drop in antibody levels was observed. In Malay population, drastic fall of antibody response was observed after W16 in compared to the Chinese and Indian populations (Fig. 2A). The seroconversion of anti-S IgG antibody in Malay population (536 BAU/ml) was fivefold higher than the threshold antibody level, whereas the seroconversion of anti-S IgG in Indian and Chinese populations (1007 BAU/ml and 982 BAU/ml, respectively) was found to be nearly nine-fold higher than that of the positive threshold (109 BAU/ml) following W24 of second Comirnaty vaccine (Fig. 2A and B) dose. Merely two (1.4%) and four individuals (2.9%) respectively exhibited an anti-S IgG antibody response below the threshold value at W20 and W24 after 2nd Comirnaty vaccination response (Fig. 2B).

Unlike Comirnaty vaccination, recipients of either Vaxzevria or CoronaVac acquired the peak anti-S IgG antibodies at W2, although the level of antibody responses was considerably lower (*P* = 0.011, Mann-Whitney U-test) than the Comirnaty vaccinated group (Fig. 2A-F). Following W2 of the second Vaxzevria vaccination, there was a significant difference (*P* = 0.007) in the anti-S IgG response in the Indian (953.3 BAU/ml), Chinese (745.5 BAU/ml), and Malay (590 BAU/ml) ethnic groups (Fig. 2C). Compared to the Indian and Malay populations, the Chinese population had a substantially lower anti-S IgG response at W4 following Vaxzevria immunisation (*P* = 0.002). The anti-S IgG antibody was subsequently reduced in all ethnic groups after W8.

Nevertheless, W24 following the second Vaxzevria vaccination, the Malay population’s mean plasma antibody concentration was 102 BAU/ml, slightly below the threshold antibody level, while the Chinese and Indian populations’ mean anti-S IgG antibody were estimated to be 170 BAU/ml and 120 BAU/ml, respectively, just above the positive cut off (Fig. 2C). Anti-S IgG scatter diagrams after the second dosage of Vaxzevria showed that, at W20 and W24, 8.6% and 12.1% of recipients, respectively, had anti-S IgG levels below the positive threshold value. The majority of these recipients belonged to Malay population (Fig. 2D).

After W2 of the second dosage of the CoronaVac vaccination, the mean anti-S IgG antibody in the Malay recipients (695 BAU/ml) was substantially lower (*P* = 0.006) than the Chinese (945.5 BAU/ml) and Indian (886.3 BAU/ml) population (Fig. 2E). Irrespective of ethnicity, anti-S IgG antibody was drastically fall after the W4 of 2nd dose of CoronaVac vaccine. The mean anti-S IgG in Malay, Chinese, and Indians was significantly reduced at W16 following the second dose of the CoronaVac vaccination to only 86, 104, and 68 BAU/ml, respectively. This was well below the positive threshold of antibody response, and it was further lowered at W20 and W24. Following W24, the anti-S IgG antibody response in Malay, Chinese, and Indian populations was estimated as low as 26.8, 38, and 34.5 BAU/ml, respectively (Fig. 2E). The scatter diagram following second dose of the CoronaVac demonstrated that following W16, W20, and W24; 54.3%, 65.2%, and 84.8% recipients, respectively, exhibited anti-S IgG below the positive threshold (Fig. 2F).

**Fig. 3:**
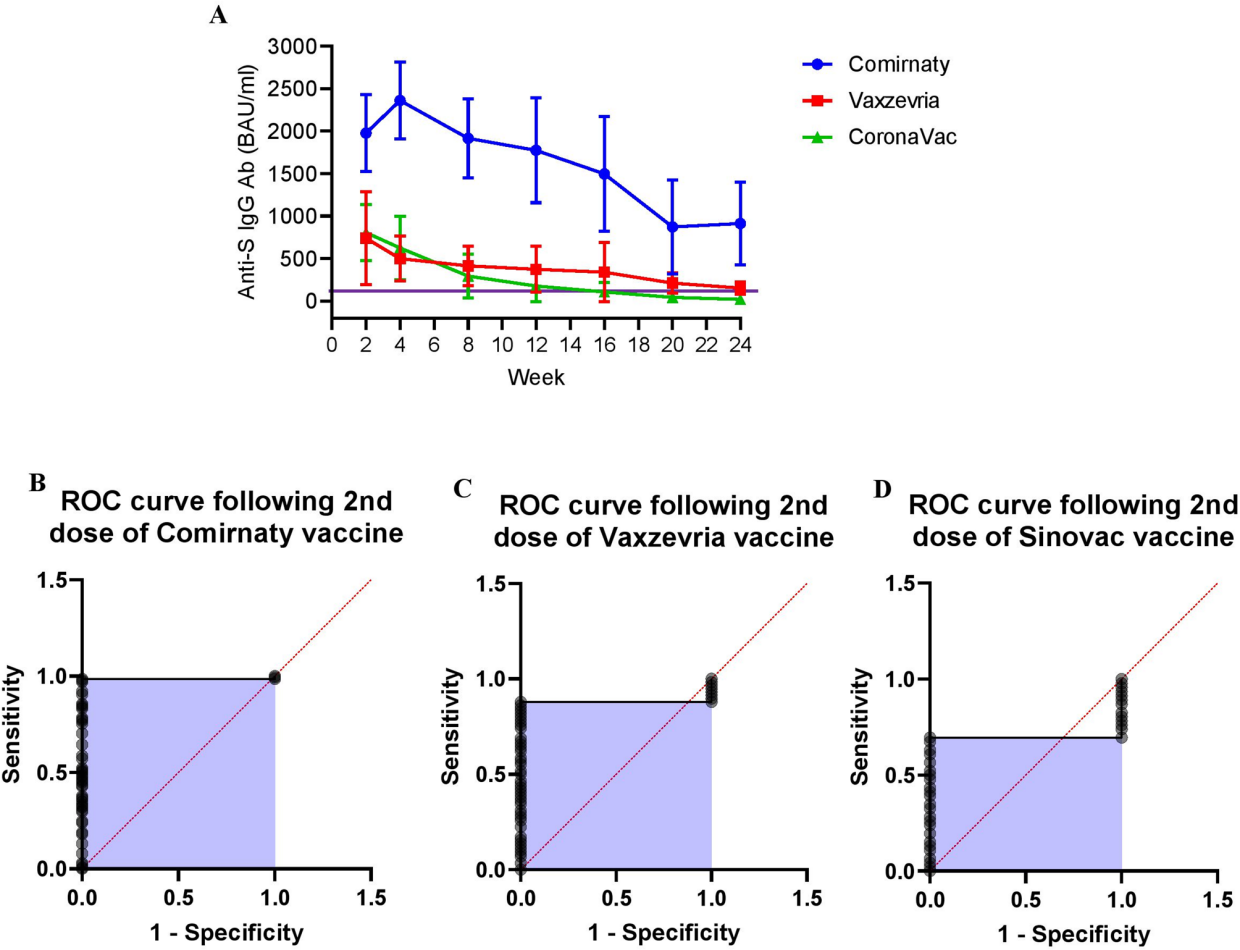
(**A**) Area under the curve (AUC) response following Comirnaty, Vaxzevria and CoronaVac vaccine: Area under the curve line diagram over 24 week period following COVID-19 vaccination. The baseline for AUC was established at 109, as 109 BAU/ml indicated the positive threshold for anti-S IgG antibody. It was marked as the purple line. The area under Comirnaty titre was significantly higher in compare to Vaxzevria and CoronaVac (*P* < 0.0001). In the analysis of Comirnaty and Vaxzevria over the specified time frame, a positive peak was observed in all areas. Conversely, in the case of CoronaVac, area of negative peak was noted after week 16. (**B**) Receiver operating characteristic (ROC) curves of anti-S IgG response following 24 weeks of comirnaty vaccine. (**C**) Receiver operating characteristic (ROC) curves of anti-S IgG response following 24 weeks of Vaxzevria vaccine. (**D**) Receiver operating characteristic (ROC) curves of anti-S IgG response following 16 weeks of CoronaVac Vaccine.

Area under curve (AUC) analysis among the three vaccines over time clearly demonstrated that anti-S IgG seroconversion was significantly higher in Comirnaty vaccine group (*P* < 0001), while recipients of CoronaVac prominently demonstrated the area of negative peak after W16 of second vaccine dose (Fig. 3A). Receiver operating characteristic (ROC) curves of anti-S IgG response following different vaccination clearly demonstrated that recipients of Comirnaty (area under the ROC curve was 0.9855 (95% CI 0.9656-1.000) *P* < 0001) (Fig. 3B) and Vaxzevria area under the ROC curve was 0.8793 (95% CI 0.7955–0.9613) *P* < 0001) (Fig. 3C) vaccines developed highly sensitive and specific anti-S IgG antibody even after 24 weeks of vaccination. In contrast following 16 weeks of CoronaVac vaccine anti-S IgG antibody sensitivity was drastically reduced (area under the ROC curve was 0.696 (95% CI 0.563–0.829) *P* = 0.0012) (Fig. 3D).

### Anti-S IgG seroconversion and BMI

**Fig. 4:**
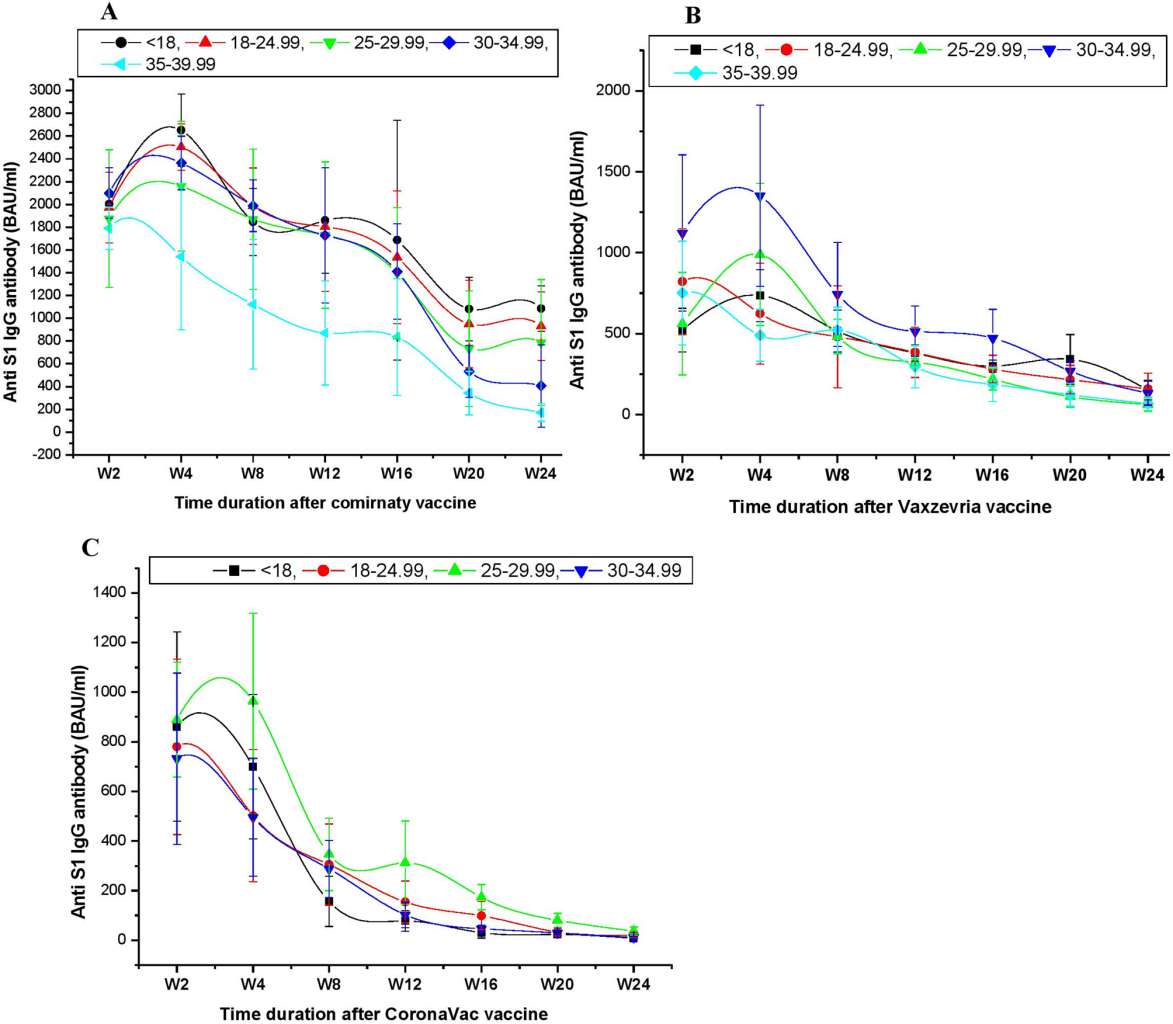
Anti-S IgG kinetics and its relation to BMI. Following WHO guideline BMI was categorized as following. BMI < 18–Underweight, BMI < 18–24.99—Normal, BMI < 25-29.99- Overweight, BMI < 30–34.99—class I obese, BMI < 35–39.99—Class II obese. (**A**) Anti-S IgG kinetics in relation to BMI following Comirnaty vaccine. Seroconversion of anti-S IgG response across all time line was observed much higher in underweight and normal BMI group in compare to overweight or class I and class II obese group. (**B**) Anti-S IgG kinetics in relation to BMI following Vaxzevria vaccine Seroconversion of anti-S IgG response of class I obese and Overweight were much potent across all the time line in compare to normal BMI and underweight. (**C**) Anti Anti-S IgG kinetics in relation to BMI following CoronaVac vaccine. Seroconversion of anti-S IgG response across all time line was observed much higher in overweight and underweight group in compare to normal or class I obesity.

In Comirnaty group, peak of anti-S IgG was observed at W4 in underweight (2651.7 BAU/ml) and normal BMI individual (2503.2 BAU/ml) which was significantly lower in class II obese group (1541 BAU/ml) (*P* = 0.002). Moderate to high anti-S IgG was observed in overweight (2161.6 BAU/ml) and class I obese group (2364 BAU/ml). The class I and class II obese groups showed a significant and sharp drop in anti-S IgG following W16. Anti-S IgG was found to be ten and nine-fold greater from its positive threshold at W24, respectively, in the underweight and normal BMI groups. In contrast, anti-S IgG was only 3.7 and a 1.5-fold greater than its positive control in the class I and class II obese groups (Fig. 4A).

The peak of anti-S IgG in the Vaxzevria group was seen at W4 in class I obese group (1352 BAU/ml) and overweight group (989.2 BAU/ml), however it was significantly lower in normal BMI (624.6 BAU/ml) and underweight group (736 BAU/ml) (*P* = 0.0072). Slow decline of anti-S IgG was observed across all BMI group following W16. At W24, the normal BMI, underweight, and overweight class I groups’ anti-S IgG were 1.5, 1.4, and 1.2 times higher than the positive threshold, respectively, whereas the overweight and class II group’s anti-S IgG were slightly below the positive threshold (Fig. 4B).

With the exception of the overweight group, which showed a substantially high peak (963.5 BAU/ml) at W4 (*P* < 0.01), the CoronaVac group’s anti-S IgG peak was observed at W2 in the underweight group (861 BAU/ml), normal BMI group (780 BAU/ml), and class I obesity group (732 BAU/ml). Sharp decline of anti-S IgG was observed across all BMI group following W4. All other BMI groups showed below-positive threshold anti-S IgG at W16, with the exception of the overweight group (173.2 BAU/ml) and the normal BMI group (109.8 BAU/ml) (Fig. 4C). In multivariate logistic-regression equation after adjustment of age, gender and ethnicity, the recipient (normal and underweight) with Comirnaty vaccine was substantially more likely to have an anti-S IgG > 109 BAU/ml (odds ratio 91.4; *P* < 0.0001) following W24 of second dose vaccination. Recipients with class I and class II obesity had lesser antibody responses to the Comirnaty vaccination than did those who were normal or underweight. Class I obesity, as well as those who were normal or underweight, were linked to stronger humoral immune responses to the Vaxzevria vaccine.

### Gender, ethnicity and age stratification with anti-S IgG antibody following vaccination

Overall vaccine exposure and plasma anti-S IgG antibody of outcomes following dose 2 vaccination were found statistically significant (*P* < 0.05) (Table 3). Both male and female genders demonstrated a strong correlation between plasma anti-S IgG antibody and vaccine exposure (males *P* < 0.0001, females *P* < 0.0001). Similarly, we observed there was a strong association between plasma anti-S IgG antibody and vaccine exposure among three different ethnicities of Malaysia (Malay *P* < 0.0001, Chinese *P* < 0.0001 and Indian *P* < 0.0001). We also observed that both young adults (18–24 years) and older individuals (25 years and above) demonstrated a significant correlation between plasma anti-S IgG antibody and vaccine exposure (young adults *P* < 0.0001, older individual. *P* < 0.0001). Therefore, gender, ethnicity and age were not found as a confounding factor for the production plasma anti-S IgG following 2nd dose of vaccination. Multivariate logistic model after adjusted for age, gender, ethnicity and having comorbidities such as hypertension, diabetes mellitus, overweight, chronic respiratory disease, coronary artery diseases and liver disease CoronaVac was producing significantly (*P* = 0.002) lower plasma anti-S IgG antibody following 20 weeks of 2nd dose of vaccination. It showed that wanning of plasma anti-S IgG antibody was much faster in CoronaVac group in compare to Comirnaty of Vaxzevria vaccine.

**Table 3:** Gender, ethnicity and age stratification for association between different types of vaccines and anti-S IgG antibody level following 24 weeks of vaccination.

Exposure	Having anti-S IgG antibody above positive cut-off		Chi-square	*P* value
	Yes, N	No, N		
Overall vaccine exposure and outcomes of anti-S IgG antibody level (after Dose 2)				
Comirnaty	134	4	144.6	< 0.0001
Vaxzevria	51	7		
CoronaVac	7	39		
Male gender stratified for vaccine exposure and outcomes of anti-S IgG antibody				
Comirnaty	42	2	40.9	< 0.0001
Vaxzevria	18	1		
CoronaVac	4	12		
Female gender stratified for vaccine exposure and outcomes of anti-S IgG antibody				
Comirnaty	92	2	105.3	< 0.0001
Vaxzevria	33	6		
CoronaVac	3	27		
Malay ethnicity stratified for vaccine exposure and outcomes of anti-S IgG antibody				
Comirnaty	28	2	29.2	< 0.0001
Vaxzevria	8	1		
CoronaVac	1	9		
Chinese ethnicity stratified for vaccine exposure and outcomes of anti-S IgG antibody				
Comirnaty	50	0	69.3	< 0.0001
Vaxzevria	19	1		
CoronaVac	4	20		
Indian ethnicity stratified for vaccine exposure and outcomes of anti-S IgG antibody				
Comirnaty	56	2	44.6	< 0.0001
Vaxzevria	24	5		
CoronaVac	2	10		
Age (18–24 years) stratified for vaccine exposure and outcomes of anti-S IgG antibody				
Comirnaty	101	1	110.7	< 0.0001
Vaxzevria	35	7		
CoronaVac	3	25		
Age (25 years and above) stratified for vaccine exposure and outcomes of anti-S IgG antibody				
Comirnaty	33	3	38.1	< 0.0001
Vaxzevria	16	0		
CoronaVac	4	14		

*P* value was calculated using chi-square test.

### Comparative Ig-RBD neutralizing antibody response

We have performed the SARS-CoV-2 Surrogate Virus Neutralization Test (sVNT) against the novel B.1.1.529 omicron strain from South Africa which gives us the idea about vaccines cross protection against the real-world scenario. The expression of result was percentage (%) of inhibition of Ig-RBD binding on ACE_2_ receptor using sera of Comirnaty, Vaxzevria, and CoronaVac vaccination group. After W2 of second vaccine dose, the Comirnaty (93.5%) and Vaxzevria (89.6%) groups exhibited significantly (*P* < 0.0001) higher % of inhibition and considered as high neutralization while CoronaVac group showed moderate neutralization with 63% inhibition (Table 4). The highest % of inhibition for Comirnaty (98.5%) and CoronaVac (78.2%) was observed in W4 while Vaxzevria (98.3%) was recorded in W8. Till W24 the recipients of Comirnaty showed high neutralization with lowest % inhibition of 91.3% at W24 (Table 4). Recipients of Vaxzevria demonstrated high neutralization until W16, % of inhibition subsequently decreased to 87.9% and 84.5% respectively in W20 and W24. In contrast recipients of CoronaVac showed moderate neutralization till W8 followed by weak neutralization till W16. Thereafter, % of inhibition dropped as low as 13.0% in W24 and considered as no neutralization (Table 4). Mann-Whitney U test revealed a statistically significant difference (*P* < 0.01) in neutralizing antibody levels between Comirnaty and CoronaVac recipients and between Vaxzevria and CoronaVac recipients. Neutralizing antibodies were found to be highest in the Chinese population followed by Indian and Malay population (Supplementary Table 1).

**Table 4:** Percentage (%) of Inhibition of Ig-RBD binding on ACE_2_ receptor.

Type of vaccine	Total no:	Percent of inhibition by serum Ig-RBD neutralizing antibody						
		W2	W4	W8	W12	W16	W20	W24
Comirnaty–Comirnaty	138	93.5%	98.6%	98.6%	97.8%	95.7%	92.8%	91.3%
Vaxzevria–Vaxzevria	58	89.7%	96.6%	98.3%	93.1%	93.1%	87.9%	84.5%
CoronaVac–CoronaVac	46	63.0%	78.3%	65.2%	54.3%	41.3%	28.3%	13.0%

Here W is denoted as Week.

### Association of anti-S IgG and Ig-RBD neutralizing antibody

Our findings indicated a monotonic relationship between an anti-S IgG and % inhibition of Ig-RBD binding on ACE_2_ receptor after vaccination. The duration-dependent anti-S IgG seroconversion and the percentage of neutralization after the second doses of the Comirnaty and Vaxzevria vaccines, respectively, were found to be highly (*P* = 0.005) correlated (Spearman correlation coefficients (r_s_) were 0.9045 and 0.9009, respectively). In contrast, anti-S IgG and the percentage of neutralization after the CoronaVac vaccine did not significantly correlate (*P* = 0.0676) (r_s_=0.7207).

## Discussion

In order to lessen the COVID-19 pandemic’s worldwide effects, vaccines have been developed and distributed quickly throughout the globe. Like many other nations, Malaysia had used a variety of COVID-19 vaccines as part of a national immunisation program. The introduction of numerous vaccines, such as CoronaVac, Vaxzevria, and Comirnaty, had made it imperative to research the relative efficacy of these shots in different populations, particularly the most vulnerable healthcare professionals and medical students across Malaysia. Numerous factors, such as age, sex, pre-existing medical conditions, and genetics, might affect an antibody’s response to a vaccination^33,34^. This comprehensive research provided a thorough duration-dependent comparison of the humoral immunity (antibody) kinetics to the Comirnaty, Vaxzevria, and CoronaVac vaccines across Malaysia’s diverse ethnic communities.

Comirnaty, an mRNA-based vaccination that encoded the spike (S) protein of SARS-CoV-2, stimulated the generation of neutralizing antibodies and activated cellular immunity, eliciting a potent immunological response^12,14^. Neutralizing antibodies were the type of antibodies associated with immunity against infection; they attached to the spike protein of the SARS-CoV-2 virus and prevented it from infecting cells^35^. Furthermore, recent research had revealed that the amount of neutralizing antibodies produced by vaccinations or spontaneous infections may wane over the course of few months to a year^36^. Among Malaysia’s three ethnic groups, we found that Comirnaty frequently produces the highest plasma Ig-RBD neutralizing and anti-S IgG antibody responses. Higher antibody titres were purportedly associated with improved defence against COVID-19 infection and protection against mortality^37^. Irrespective of ethnicity, the subsequent decline of anti-S IgG antibody after Comirnaty vaccine was observed after W16. However, compared to Vaxzevria and CoronaVac, the rate of antibody fading even after W24 was significantly lower, indicating sustained protection after Comirnaty vaccination. These findings were consistent with other results from Chile, Israel, and other regions of the world^38–40^. Several countries from European Union have also reported on the subsequent waning pattern of anti-S IgG over time^41–43^. Although the amount of neutralizing antibody was slightly higher in the Comirnaty group, the production of neutralizing antibodies following W24 of the Comirnaty and Vaxzevria vaccines were consistently maintained, which was in line with earlier findings from Southeast Asia, Brazil, South Africa, and the UK^14,35,36,43,44^. Neutralizing antibody was drastically reduced over 24 weeks of CoronaVac vaccine. Even after 16 weeks of CoronaVac vaccination, the area under the ROC in response to ant-S IgG kinetics after vaccination made it abundantly evident that the sensitivity was significantly lower than with Comirnaty and Vaxzevria. Duration-dependent area under the curve analysis, as well as ROC, clearly revealed that recipients of the CoronaVac vaccine had a higher likelihood of wanning of neutralizing and anti-S IgG antibody much earlier than the recipients of the Comirnaty and Vaxzevria vaccine. A number of countries around the world had previously reported a rapid decline in serum Ig-RBD after W16 of the CoronaVac immunization^15,45–47^. In contrast to inactivated viral vaccine (CoronaVac), both mRNA (Comirnaty) and adenovirus vector (Vaxzevria) vaccines activate longer-lived memory B cells and CD4^+^ T cells, which ultimately evoke the production of serum anti-S IgA and anti-S IgG antibodies and Ig-RBD neutralizing antibodies^48^. Comirnaty (mRNA) vaccine was internalized and quickly translated by antigen-presenting cells (APC), where it triggered strong humoral immune as well as adaptive responses by activating more IgG specific memory B cells and CD8 + T-Cells thereby generating more Ig-RBD neutralizing antibodies^49^. In contrast recombinant non-replicating Vaxzevria was a viral vector vaccine that leads to host cells expressing the S protein thus stimulating a strong humoral and cell-mediated immune response^50^.

A person’s response to vaccinations could also be influenced by number of factors including their ethnicity because of variations in immunity, life-style, exposure to environment, and even food habit^33^. Following Comirnaty and the CoronaVac vaccination, we found the peak anti-S IgG responses were higher in the Chinese population. Neutralizing antibodies were found to be highest in the Chinese population even after 24 weeks of Comirnaty and Vaxzevria vaccination. Regardless of the vaccine type, Chinese people in Malaysia showed higher antibody titre than other ethnic groups. Research from China and other East-Asian nations had shown that Chinese people respond more strongly to mRNA vaccinations than to Vaxzevria and CoronaVac in terms of antibody production^37,51^. This might be explained by their genetic complexity, 5life-style, or food habit^51,52^. The highest peak anti-S IgG antibody responses were found in the Indian population following vaccination with Vaxzevria at W2. The Indian population, across all ethnic groups, has significantly greater anti-S IgG antibody levels up until W12. Prior research from India suggests that the Indian people may have a more robust antibody response to single recombinant vaccine likeVaxzevria^53,54^.

Compared to Chinese or Indian populations, Malay populations showed comparatively lower percentage of neutralization and anti-S IgG responses following the Comirnaty vaccine after W16. Indian and Chinese populations had 1.87and 1.67-fold higher anti-S IgG at W24, respectively, than did the Malay group. Furthermore, anti-S IgG in Malay population sharply decreased following vaccinations with Comirnaty at W16 and with Vaxzevria and CoronaVac at W8. Regardless of the vaccine type, the Malay population’s antibody response was comparatively lower than that of the Chinese and Indian populations. While there are differences in humoral immune response to different vaccines among Malaysia’s three ethnic groups, our findings demonstrated that age, gender, and ethnicity did not function as confounding factors in evoking humoral immune kinetics.

This could be attributed due to other confounding factors related to type of vaccine and lifestyle including BMI^52,55^. Majority (47.91%) of overweight and obese individuals belonged to Malay population. We observed that recipients with higher BMI had reduced immune response to Comirnaty vaccine than those with normal BMI or underweight. This was consistent with previous studies showing that a high BMI reduces immune response, by decreased production and activity of plasma B cells, Th2 cells, and CD8 + memory T lymphocytes^56,57^. Watanabe et al. reported that people with high BMI have lower immune responses to mRNA vaccinations, which was consistent with our findings^58^.

However, higher BMI was linked to stronger immune responses to non-mRNA vaccines such as Vaxzevria. Elevated leptin levels in patients with high BMI contribute to pro-inflammatory states and increased immune cell activation, which may improve the efficiency of vaccines^59^. These findings highlight the intricate relationship between BMI and vaccine induced humoral immunity.

There were certain limitations to our investigation. A more thorough investigation is needed to examine the differences in antibody response between various ethnic groups.

Second, the majority of recipients were between the ages of 20 and 45, with fewer those over 45. Consequently, we are unsure if the effect of vaccine’s impact on the elderly is overstated or understated. Precise role of BMI and its association with humoral and cell mediated immunity following vaccination.

## Conclusion

In conclusion, Comirnaty vaccination elicited a far superior duration-dependent humoral-immune response, regardless of ethnicity. Comirnaty and Vaxzevria exhibited prolonged Ig-RBD neutralizing activity in compare to CoronaVac. BMI may play a crucial role in developing humoral immune response following vaccination. The Chinese and Indian populations may have a better antibody response than the Malay population after vaccination. Further research would be necessary to determine the precise function of BMI and ethnicity in eliciting the humoral immune response over the long run.

## Electronic supplementary material

Below is the link to the electronic supplementary material.

Supplementary Material 1

## Data Availability

According to institutional regulation of ethics committee, data representing sensitive patient information are restricted; our data involving clinical participants are not freely available in a public repository. However, data are available upon request to the corresponding author Dr Sabyasachi Das.
