# Supplementary material for: Comparative analysis of humoral immunity kinetics following three COVID-19 vaccines in a multi-ethnic cohort of medical students and healthcare professionals across Malaysia
Source: Sci Rep. 2025 Jul 1;15:21953. doi: 10.1038/s41598-025-07895-6 (PMC12216813; doi:10.1038/s41598-025-07895-6)
Supplement: Supplementary file 1 — Supplementary Material 1 [file 41598_2025_7895_MOESM1_ESM.doc]

**Supplementary Methods**

1. Body Mass Index
   An electronic weighing scale was employed to evaluate individual body weight during a four-week follow-up, with participants attired in loose-fitting clothing and without footwear. A "drop-down" tape was employed to ascertain height. The weight (kg) of each participant was divided by the square of their height (m²) to calculate their BMI.^1^
2. Fasting Blood Glucose and HbA1c
   Venipuncture was performed to collect 5 ml of venous blood after a 12-hour fasting period. Blood samples were collected in a fluoride oxalate-coated vacutainer tube both prior to (week 0) and after to the completion week 24 os study. Blood samples were centrifuged at 3000 RPM for 15 minutes at 10°C to obtain the plasma. Plasma was thereafter stored at -75°C until needed. Fasting blood sugar (FBS) was quantified biochemically utilizing a Shimadzu spectrophotometer. HbA1c was measured using an automated HbA1c analyzer as previously detaile.^2^
3. Blood pressure

Blood pressure was measured through digital sphygmomanometer as describe by the manufacturer.

1. Lipid Profile:

Total cholesterol (TC), triglycerides (TG), and high-density lipoprotein (HDL) cholesterol were enzymatically quantified using a Shimadzu autoanalyzer.^3^ The Friedewald formula [LDL = TC–HDL– (TG/5)] was employed to compute LDL when the TG level was below 4.6 mmol/L, as previously detailed.^4^

**Reference:**

1. Zuo L et al. Comparison of High-Protein, Intermittent Fasting Low-Calorie Diet and Heart Healthy Diet for Vascular Health of the Obese. *Front Physiol* 2016; 7: 350.
2. Pilla R,et al. Glycated Haemoglobin (HbA1c) Variations in Nondiabetics With Nutritional Anemia. *Cureus*. 2020; 12(11): e11479.
3. Doran B, et al. Bangalore S. Prognostic value of fasting versus nonfasting low-density lipoprotein cholesterol levels on long-term mortality: insight from the National Health and Nutrition Examination Survey III (NHANES-III). Circulation. 2014; 130: 546-53.
4. Friedewald WT, Levy RI, Fredrickson DS. Estimation of the concentration of low-density lipoprotein cholesterol in plasma, without use of the preparative ultracentrifuge. *Clin Chem* 1972; 18(6): 499-502.

**Supplementary Table 1**: Association between anti-S IgG seroconversion and percent neutralization following Comirnaty, Vaxzevria and CoronaVac vaccination

.

S-IgG (%) represented the Percentage (%) of recipients with seropositive anti-S IgG antibody. Neutralizing Ab (%) represented the percentage (%) of viral growth inhibition.

|  | Anti-S IgG (%) | Comirnaty - Comirnaty Neutralizing Ab  (% inhibition) | | | Anti-S IgG | Vaxzevria - Vaxzevria Neutralizing Ab (% inhibition) | | | Anti-S IgG | CoronaVac- CoronaVac Neutralizing Ab  (% inhibition) | | |
| --- | --- | --- | --- | --- | --- | --- | --- | --- | --- | --- | --- | --- |
|  |  | Malay | Chinese | Indian |  | Malay | Chinese | Indian |  | Malay | Chinese | Indian |
| W2 | 100 | 90.5 | 96 | 94.5 | 94.83 | 87 | 91 | 90 | 100 | 63 | 64 | 62 |
| W4 | 100 | 96 | 100 | 100 | 100 | 95.5 | 96.5 | 97.5 | 100 | 77 | 80 | 80 |
| W8 | 100 | 98 | 98 | 99 | 98.28 | 97 | 96.5 | 97.5 | 86.95 | 65 | 66 | 62.5 |
| W12 | 100 | 97 | 98 | 97 | 98.28 | 94 | 94.5 | 92 | 68.43 | 52 | 57 | 56 |
| W16 | 99.27 | 95 | 95 | 96. | 93.1 | 92,5 | 93.5 | 93 | 46.30 | 40.5 | 40 | 42.5 |
| W20 | 98.55 | 92.5 | 94 | 93 | 91.37 | 85 | 90 | 91 | 33.71 | 27 | 28 | 29 |
| W24 | 98.55 | 88.5 | 92.5 | 93 | 87.93 | 82 | 86 | 84 | 22.8 | 18 | 21 | 22.5 |
